# Editorial: Immune-mediated lung injury

**DOI:** 10.3389/fmed.2023.1292074

**Published:** 2023-11-03

**Authors:** Theodoros Karampitsakos, Paolo Spagnolo, Argyris Tzouvelekis

**Affiliations:** ^1^Division of Pulmonary, Critical Care and Sleep Medicine, Ubben Center for Pulmonary Fibrosis Research, Department of Internal Medicine, Morsani College of Medicine, University of South Florida, Tampa, FL, United States; ^2^Respiratory Disease Unit, Department of Cardiac, Thoracic, Vascular Sciences and Public Health, University of Padova, Padova, Italy; ^3^Department of Respiratory Medicine, University Hospital of Patras, Patras, Greece

**Keywords:** post-COVID, long-COVID, post-COVID-19-interstitial lung disease, idiopathic pulmonary fibrosis, immunity, monocytes

## The dawn of a new immune-mediated lung disease: post-COVID-19 interstitial lung disease

The role of immunity in acute and chronic lung injury has been now established (Sweis et al.) (1, 2). With regards to interstitial lung diseases (ILDs), a possible association with immunity was first reported almost 50 years ago. In particular, Crystal et al. reported that “The fibrotic process is probably irreversible, however the inflammatory and immune processes causing it may be amenable to therapy if diagnosed early” (3). Despite further reports linking immune deregulation and chronic lung injury, the role of immunity had been severely underscored in the past mainly due to the disappointing results of immunosuppressive and immunomodulatory agents such as corticosteroids in patients with idiopathic pulmonary fibrosis (IPF) (4). Most recently, the interest has been revived. Clinical and translational observations fueled mechanistic discoveries on the role of immunity in lung injury. For example, cellular deconvolution of the 52-gene signature, a highly reproducible biomarker in IPF, showed that monocytes are the cellular source of the upregulated genes. This paved the way for clinical studies showing the prognostic potential of monocyte count in ILDs as well as mechanistic studies investigating the role of monocytes/ myeloid derived suppressor cells in pulmonary fibrosis (5–9).

Given the increased interest for the association of immunity with lung injury, this Research Topic included articles presenting immune insights in the context of IPF as well as articles highlighting the impact of ILD in patients with connective tissue diseases such as scleroderma and myositis (Kirgou et al.; Liossis and Bounia; Liossis and Staveri; Karampitsakos et al.). Importantly, this Research Topic highlighted the dawn of a new immune-mediated lung disease, named post-COVID-19-ILD (Bernardinello et al.; Karampitsakos et al.). In particular, data from the Greek registry of patients with post-COVID-19-ILD showed that Forced Vital Capacity% predicted and Diffusing capacity for carbon monoxide% predicted were below 80% in 25.8 and 30.6% of patients in the 3-month follow up, respectively (Karampitsakos et al.). Of note, 5.6% of patients presented with “fibrotic-like” changes and persistent functional impairment at the 6-month follow-up leading thus to implementation of antifibrotics. Similarly, an Italian study demonstrated that 6.9% of the cohort had not recovered in terms of lung disease in the 1-year follow-up (Bernardinello et al.). Patients that did not recover in the 1-year follow-up were older, more frequently current smokers and had worse PaO_2_/FiO_2_ on admission at the time point of hospitalization compared to patients that recovered (Bernardinello et al.). Given that both studies showed that a (not negligible) minority of patients with COVID-19 exhibit persistent lung disease even 1 year following acute infection, questions about the long-term trajectory of these patients arise. Taking into consideration that COVID-19 and fibrotic-ILDs have (1) common radiographic features and (2) common innate and adaptive immune responses [the aforementioned gene-signature that predicted outcomes in IPF, predicted outcomes in COVID-19, as well (10)], the following remain to be addressed:

1. Are “fibrotic-like” changes in patients with post-COVID-19 reversible? Does radiologic fibrosis necessarily mean histologic fibrosis? Will the treatment of “immature fibrosis” prevent irreversible disease or radiographic findings will resolve/not progress irrespective of treatment? Ongoing studies will hopefully shed light to these questions.
2. Are genes that predict mortality in IPF and COVID-19, still abundantly expressed in post-COVID-19-ILD? Extensive investigation of genes that predict mortality in IPF suggested that monocytes/myeloid derived suppressor cells persist during the disease course, while T cells might exhibit exhaustion (5–11). Similarly in COVID-19, myeloid cells have been shown to be highly activated (12), with dysfunctional HLA-DR^lo^CD163^hi^ and HLA-DR^lo^S100A^hi^ CD14^+^ monocytes being present in patients with severe disease (13). Moreover, T cell subpopulations of patients with SARS-CoV-2 infection had exhaustion features (14– 16).
  However, further data are needed to understand if the aforementioned phenomenon persists in post-COVID-19-ILD. If not, a less abundant expression of genes that are directly related with monocytes, might mean a gradual “immune recovery” in post-COVID-19-ILD and probably a favorable long-term course. Contrary to IPF, persistence of monocytes and impaired T cell response might not be the case in post-COVID-19-ILD, as the epithelial injury happened only in the acute phase of infection and is not repetitive (17, 18). Studies implementing single-cell RNA-sequencing to compare post-COVID-19-ILD and IPF could hopefully address this unmet need and help clinicians “predict” the long-term outcomes of patients with post-COVID-19-ILD.
  Addressing the two aforementioned questions, will substantially contribute to the management of this new entity with unknown long-term consequences. Despite that the acute phase of the pandemic is thankfully over, clinicians should not forget that a minority of COVID-19 survivors have persistent lung disease. Timely and appropriate management of this new entity might positively impact patients' health-related quality of life on a long-term basis.

## Author contributions

TK: Conceptualization, Investigation, Writing—original draft. PS: Conceptualization, Investigation, Writing—original draft. AT: Conceptualization, Investigation, Writing—original draft.
